# Acute Right-Sided Abdominal Pain in an Adolescent: Expect the Unexpected

**DOI:** 10.7759/cureus.16649

**Published:** 2021-07-26

**Authors:** Somaya Abuelazm, Harsha Kanumalla, Marvic Taborda-Alvarez, Jacob Eisdorfer, Ratna Basak

**Affiliations:** 1 Pediatrics, Brookdale University Hospital Medical Center, New York City, USA; 2 Colorectal Surgery, Brookdale University Hospital Medical Center, New York City, USA

**Keywords:** cecal diverticulitis, adolescent, pediatrics, gastroenterology, abdominal pain, surgery, general surgery, pediatric surgery, appendicitis, pediatric lap. surgery

## Abstract

Acute right lower quadrant (RLQ) abdominal pain is a common presenting complaint in the emergency department (ED). The most common cause is acute appendicitis, generally requiring surgical intervention. We present a rare cause of RLQ abdominal pain mimicking acute appendicitis.

## Introduction

A 16-year-old male presented to the emergency department (ED) with right lower quadrant (RLQ) abdominal pain for two days. Contrast-enhanced computed tomography (CECT) scan was read as a significantly thickened appendix with peri-appendiceal fat stranding that could be due to appendicitis. The patient was taken for a laparoscopic appendicectomy but intra-operatively, it was discovered to be a rare entity.

## Case presentation

A 16-year-old African American male presented to the ED with a two-day history of abdominal pain, fever, and vomiting. The pain was in the RLQ, with an intensity of 10/10 and non-radiating, aggravated by walking, and not relieved by oral analgesics. Few hours prior to the ED visit, he developed a fever (39.2°C) and a single episode of non-bilious, non-bloody, and non-projectile vomiting. There were no changes in bowel habits, dysuria, testicular pain, abdominal trauma, or previous similar episodes. He had no significant past medical, family, or surgical history. There were no known allergies or recent travel. Immunizations were up to date.

The initial physical exam showed an ill-looking adolescent, with a heart rate of 119 beats/min, with a normal temperature, respiratory rate, blood pressure, saturation, and capillary refill time. The abdominal examination showed localized tenderness at the RLQ, but no rigidity or rebound. The bowel sounds were audible. Other systemic examinations were normal.

Laboratory investigations revealed hemoglobin of 11.7 g/dL, raised white cell count (11.5 x 109/L), and neutrophils of 54%. C-reactive protein (CRP) was 5.9 mg/L. Renal function, serum amylase, and sickle cell screen were negative. Urine analysis was in the normal range and the rapid severe acute respiratory syndrome coronavirus 2 (SARS-CoV-2) test was negative. Stool examination was negative for *Clostridium difficile* and occult blood.

**Figure 1:**
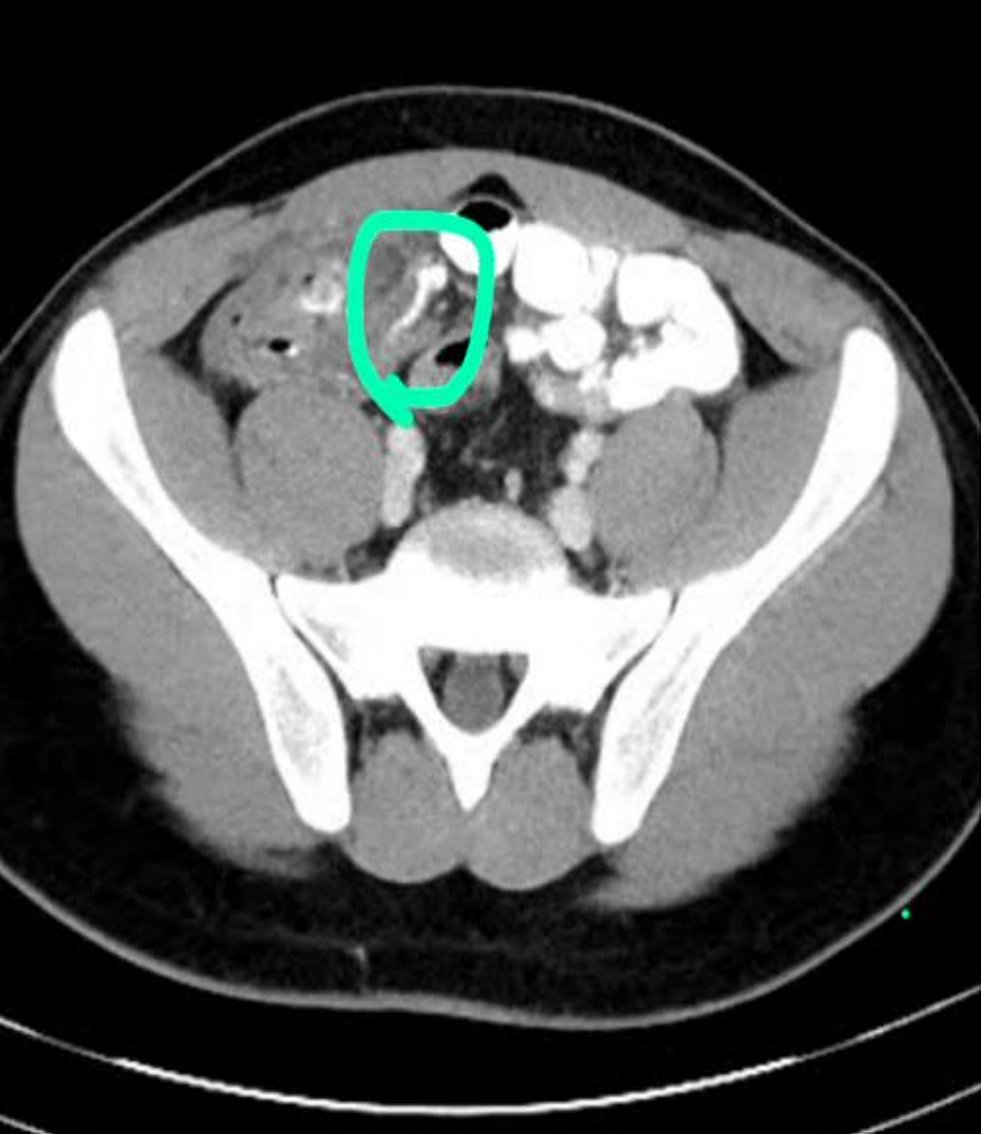
CECT scan of the abdomen-pelvis showing the appendix to be significantly thickened with peri-appendiceal fat stranding. CECT, contrast-enhanced computed tomography

Erect chest and abdominal radiographs demonstrated mildly dilated small bowel loops with air throughout the colon, but no evidence of pneumoperitoneum. A CECT scan of the abdomen-pelvis showed the appendix to be significantly thickened with peri-appendiceal stranding which could be due to acute appendicitis. There is also an adjacent wall thickening of the terminal ileum and cecum. There were no signs of perforation (Figure 1).

In the ED, the patient received analgesics, esomeprazole, ondansetron, cefotaxime, and normal saline bolus. A surgical consultation was done, and the patient was taken for laparoscopic appendectomy.

**Figure 2:**
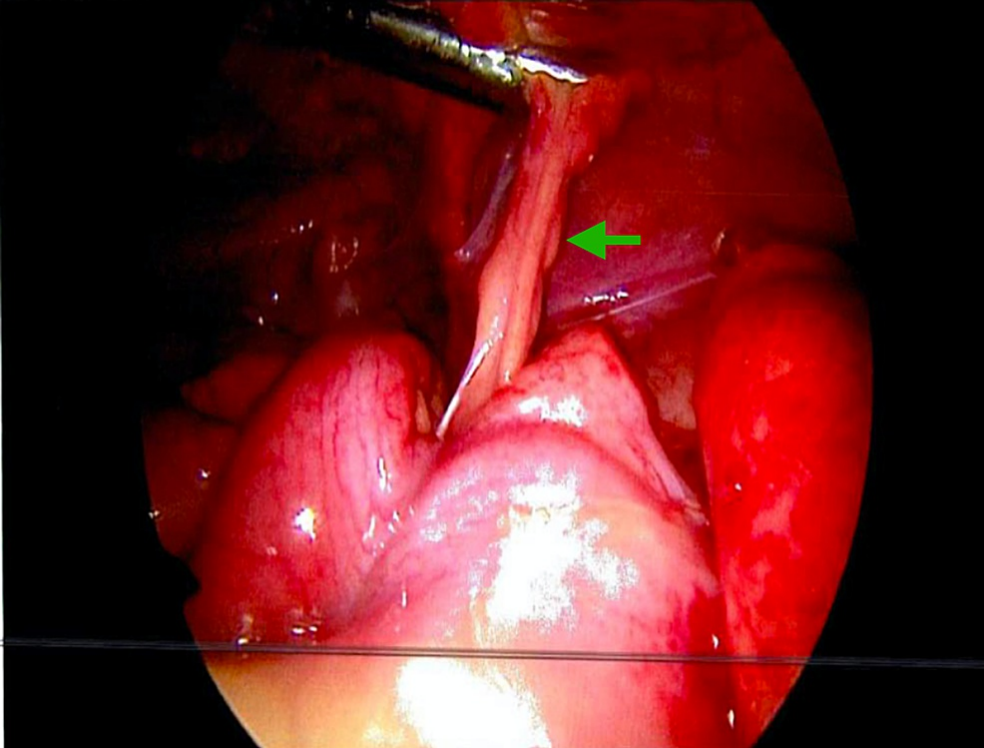
Intra-operative picture showing inflamed cecum with normal appearing appendix (green arrow).

Laparoscopy showed visible inflammation in the cecum; a non-inflamed appendix was visualized in the RLQ. The outpouching of the cecum was inflamed which seemed to be a congenital cecal diverticulum (Figure 2). A second opinion was requested from the colorectal surgeon who performed a laparoscopic Ileocecal resection (right hemicolectomy) with primary anastomosis. The patient tolerated the procedure well.

Surgical pathology of the resected tissue examination showed an enlarged inflamed cecum with fatty inflamed scattered white-tan fibrous adhesions. The lumen showed diverticulum at the cecum that was consistent with the outpouching area. The rest of the mucosa appeared grossly unremarkable; the tissue beneath the outpouching diverticulum was thickened and inflamed. The appendix appeared grossly normal.

Microscopic examination reported cecal diverticulum with acute diverticulitis and marked ulceration, suggestive of walled-off perforation; marked acute serositis with peri-colonic abscesses and edema; margins viable and 15 reactive lymph nodes with sinus histiocytosis.

Post-operatively our patient was admitted to the pediatric floor with IV hydration, antibiotics, pain medications, and enoxaparin. On the third post-operative day, he developed progressive abdominal distension with absent bowel sounds suggestive of postoperative ileus. He was kept nil orally and was placed on continuous nasogastric suction.

On day 7, due to persistent ileus with normal electrolytes, CT abdomen-pelvis with contrast was done, which showed findings concerning small bowel obstruction with a transition point in the RLQ at the enterocolic anastomosis with a small amount of scattered free fluid in the abdomen and pelvis, small bilateral pleural effusions but no definite organized/encapsulated fluid.

The conservative management continued with gentle ambulation, nil orally, and IV hydration. On the eighth day post-operative the abdominal distention improved, with the presence of bowel sounds, oral fluids were started, and he was discharged home on the 10th day post-operative with a week follow-up with the colorectal surgeon.

Ultimately, the patient was diagnosed with cecal diverticulitis with postoperative ileus.

## Discussion

Acute abdominal pain is a commonly encountered and often a challenging problem in the pediatric age group. The causes of acute abdominal pain differ according to age and can range from self-limited benign conditions to urgent medical or surgical ones.

Acute diverticulitis is an infrequent etiology and is not considered in the differential diagnosis during the assessment of an adolescent presenting with acute right-sided abdominal pain. Right-sided diverticulitis is often misdiagnosed as acute appendicitis, and a correct diagnosis is made only in 14% of patients [1].

Colonic diverticular disease is commonly encountered in the older population and right-sided diverticulosis has a higher incidence in Asian population [2]. The etiology is intestinal hypoganglionosis or aganglionosis. Diverticulosis has been related to genetic predisposition in combination with other risk factors. Additionally, the diverticular disease has been identified in young adults with cystic fibrosis and genetic syndromes affecting connective tissue; Marfan syndrome, Ehler-Danlos syndrome, and Williams Beuren syndrome [3] . The risk factors associated with the development of diverticulosis are low fiber diet, high body mass index (BMI ≥ 25), disorders affecting the colonic peristalsis, and structural colonic wall anomalies [4]. A recent study showed an association between obesity and diverticulitis [5].

It is important to classify diverticulitis into complicated or uncomplicated types and to exclude other causes of abdominal pain. Complicated cases are associated with bleeding, perforation, and recurrence, and are common in adolescents and younger populations. The risk of complication increase in younger patients who present with fever (≥ 38°C) at the time of admission [6].

The management of acute diverticulitis in the young is similar to that in the older patients [7]. The diagnosis is confirmed by abdominal CT scan with oral and IV contrast which has a high sensitivity (93%-96%) and specificity (100%) [8]. It is considered as an aggressive disease in young patients and surgical treatment is recommended to prevent recurrent attacks [9]. Recent literature suggests that surgery for acute diverticulitis should be based on clinical findings, severity, age, and the number of episodes of diverticulitis [10]. Antibiotics are the mainstay of treatment for uncomplicated diverticulitis; however, the latest guidelines do not recommend routine use of antibiotics as the first line of treatment for patients with uncomplicated acute diverticulitis [11].

## Conclusions

Acute right-sided diverticulitis is an uncommon cause of abdominal pain in adolescents. It is often misdiagnosed as acute appendicitis which has a higher incidence in this age group as both conditions have similar clinical presentations. The diagnosis is made intra-operatively in many cases. The management is surgical resection of the affected segments.

## References

[1] Jensen TK, Eiholm S, Achiam MP (2013). [Caecal diverticulitis in a young woman with suspected acute appendicitis]. Ugeskr Laeger.

[2] Imaeda H, Hibi T (2018). The burden of diverticular disease and its complications: West versus East. Inflamm Intest Dis.

[3] Santin BJ, Prasad V, Caniano DA (2009). Colonic diverticulitis in adolescents: an index case and associated syndromes. Pediatr Surg Int.

[4] Matrana MR, Margolin DA (2009). Epidemiology and pathophysiology of diverticular disease. Clin Colon Rectal Surg.

[5] Nguyen GC, Sam J, Anand N (2011). Epidemiological trends and geographic variation in hospital admissions for diverticulitis in the United States. World J Gastroenterol.

[6] Schauer PR, Ramos R, Ghiatas AA, Sirinek KR (1992). Virulent diverticular disease in young obese men. Am J Surg.

[7] Pisanu A, Vacca V, Reccia I, Podda M, Uccheddu A (2013). Acute diverticulitis in the young: the same disease in a different patient. Gastroenterol Res Pract.

[8] Cho KC, Morehouse HT, Alterman DD, Thornhill BA (1990). Sigmoid diverticulitis: diagnostic role of CT -- comparison with barium enema studies. Radiology.

[9] Shah AM, Malhotra A, Patel B, Spira R, DePasquale JR, Baddoura W (2011). Acute diverticulitis in the young: a 5-year retrospective study of risk factors, clinical presentation and complications. Colorectal Dis.

[10] Hughes LE (1969). Postmortem survey of diverticular disease of the colon. I. Diverticulosis and diverticulitis. Gut.

[11] Ritz JP, Lehmann KS, Stroux A, Buhr HJ, Holmer C (2011). Sigmoid diverticulitis in young patients--a more aggressive disease than in older patients?. J Gastrointest Surg.

